# The Nurse—Lying in Chamber

**Published:** 1892-07

**Authors:** 


### THE NURSE IN THE LYING-IN CHAMBER.

Let the nurse be exceedingly cautious in her language in the lying-in chamber, remembering that the patient is unusually sensitive and is apt to reflect upon the sayings of her nurse. One thing that I would have you learn well is, whatever you may know of the doctor's imperfections, do not speak of them to your patient! More especially would I impress upon your minds as nurses the position you should occupy towards the obstetrician. When consulted by a patient who has not engaged her attendant, but relies upon her nurse for the recommendation of a physician (for often such is the case), do not say, as nurses are apt to say, "Do not employ either Dr. A. or Dr. B.," but rather leave out their names. Again, never criticise the doctor before your patient, whatever you may think. I am sure that one prime factor in the trouble that oftentimes exists in the care of a patient is brought about by indiscreetness on the part of the nurse; the nurse acting in the capacity of subordinate, for as such she is employed. See to it that you take your official orders from the doctor, and then carry them out to the letter; then if things turn out differently than they should you are clear of all responsibility in the case. But woe to her who takes an order from the doctor and then does as she chooses because she cannot see why this or that will not answer "just as well," for sooner or later she will weep and lament her own downfall. Do not tell stories in the sick chamber. Try and command the respect of your patient and her friends. Preserve your modesty in the lying-in chamber. Feel for a patient as you would feel for a mother or sister were she placed in similar circumstances to that of your patient. Do not expose a patient more than is necessary, and be extremely careful how you go about it. Do it when it is necessary in a delicate way. Watch closely each and every movement so far as is possible after the woman has been confined ; note symptoms and speak to the doctor about them. Do not regard them of too light importance to speak about, for the little things taken collectively often make the diagnosis when things go wrong with our patient. The doctor sees things he does not speak to the nurse about, and perhaps one little symptom he did not see, because not there, would be of paramount importance in the care of his patient. Be cheerful and sunny whatever may be your feelings; have a smile for your patient and she will certainly do a great deal better towards an early recovery. In other words, do not fret your patient by being yourself fretty. Accommodate yourself to your surroundings and the circumstances under which you are placed at the time. Keep cool at all times and you will avoid exciting your patient and her over-anxious husband or friends. If you are obliged to send for the doctor, do it in a quiet way, and then try and make your patient comfortable until the arrival of the doctor. Say but little ; remember, " a wise head makes a still tongue." If you have to say something, let it be said to the benefit of the patient. Calm her in times of excitement and nervous moments by assuring words and kindly deeds, and it will contribute to make you an ideal nurse. One thing I make a practise of which has repaid me for all the attention I have given it, and I would throw the mantle on your shoulders and have you wear it in your chosen profession; *i.e.*, keep the sick room clean and tidy. Let a patient who is naturally a neat housewife be sick in an untidy room, which is not properly cared for by nurse, and she will worry and fret herself to her detriment, and all the time wishing she could rise and just brush up a little. I have seen this exemplified so many times that I recognize the importance of a neat, cheerful lying-in room. I insist upon it, for I find that my patients do better in such a room. Whatever food you prepare for the patient, let it be tastefully prepared and nicely served. Do not serve too much—seeing too much food may cause distaste for it ; better serve it in smaller quantities and more frequently. Serve food as per doctor's orders and abide by them whatever over-kind friends or neighbors may send in to her. Another thing I would have you observe, and that is the admission of friends to the lying-in chamber. The question of admitting them is perplexing and often a delicate one to decide, but the nurse must be firm on this point. She may say the doctor prefers to keep our patient quiet for a few days. Any friend (not an acquaintance) will take it kindly and will not insist upon seeing the patient; you must use tact, fitting to the occasion, for the sick-room should not be made a rendezvous for gossipers who come in just for curiosity to see "the baby."

In closing, I will say that although I may not have advanced any thing new, I may have given you some guides that will point you towards success as monthly nurses. If so, my object is gained.—*The Nurse.*

